# Glycemic control and lipid outcomes in children and adolescents with type 2 diabetes

**DOI:** 10.1371/journal.pone.0219144

**Published:** 2019-07-01

**Authors:** Mary Margaret Barr, Stella Aslibekyan, Ambika P. Ashraf

**Affiliations:** 1 University of Alabama School of Medicine, University of Alabama at Birmingham, Birmingham, Alabama, United States of America; 2 Department of Epidemiology, University of Alabama at Birmingham School of Public Health, Birmingham, Alabama, United States of America; 3 Department of Pediatrics/Division of Pediatric Endocrinology and Metabolism, Children's of Alabama, University of Alabama at Birmingham, Birmingham, Alabama, United States of America; University of Massachusetts Medical School, UNITED STATES

## Abstract

**Background:**

The incidence of type 2 diabetes (T2DM) in children has increased dramatically. However, limited published information is known about the glycemic control and lipid outcomes in pediatric T2DM outside of clinical trials.

**Objectives:**

To determine the glycemic control and lipid measure outcomes at one and three- year follow-up in children with T2DM.

**Methods:**

A retrospective electronic medical record review of children with T2DM at the Children’s Hospital of Alabama over a 12-year period.

**Results:**

There were 301 patients with a diagnosis of T2DM who had a 1-year follow-up visit, of which 184 also had a 3-year follow-up. Most patients (78%) received either insulin with metformin or insulin alone at diagnosis. At one year, 37% of the cohort achieved ‘optimal glycemic control’ (HbA1C ≤6.5%) and 58% of patients achieved durable glycemic control (HbA1C ≤8%). Optimal glycemic control was seen in 48 patients at 3 years. The patients treated with insulin (alone or in combination with metformin) tended to have higher HbA1C at diagnosis, but had improved lipid and glycemic outcomes at follow-up. The group treated with insulin along with metformin had significant improvements in non-HDL, HDL and TC/HDL ratios. The effects of insulin treatment on glycemic control at 3 years were not statistically significant.

**Conclusion:**

With the current modality of treatment, only a minority of patients achieve optimal glycemic control at 1 and 3 years of follow-up. Studies are warranted to further elucidate the optimal therapies in the management of pediatric T2DM.

## Introduction

Over the past 20 years, the incidence of type 2 diabetes mellitus (T2DM) in pediatric and adolescent patients has dramatically increased [1–5], in parallel with the climbing rates of childhood and adolescent obesity. Although the current rate of pediatric obesity is not increasing, the incidence of T2DM continues to rise, suggesting especially adverse metabolic sequelae of obesity in young patients [3]. The incidence and burden of T2DM fall heavily on racial minorities in the US, as well as on individuals of lower socioeconomic status [1, 6, 7]. The complex pathophysiology and natural progression of type 2 diabetes in the pediatric population are not fully understood. Insulin resistance (IR), resulting from genetic predisposition, obesity, and pubertal hormones, plays a significant role. Continued weight gain, poor lifestyle, excessive caloric consumption, and secondary causes such as intake of drugs augment the metabolic derangements [8–13]. Patients with T2DM are known to have atherogenic dyslipidemia which is worsened by ongoing insulin resistance, glucotoxicity and lipotoxicity [14–17].

Optimal management of T2DM in children is not well established [18–20]. T2DM has been traditionally considered as an adult disorder, and not all currently available pharmacological agents or treatment strategies necessarily transfer to pediatric T2DM [21–23]. Metformin, a mild insulin sensitizer, is the only oral agent approved for use in children with T2DM [24, 25]. However, the Treatment Options for Type 2 Diabetes in Adolescents and Youth (TODAY) trial demonstrated that treatment with metformin does not lead to durable glycemic control (HbA1C ≤8%) in approximately half of the subjects [20, 26]. In the same trial, the combination of metformin with rosiglitazone similarly failed to reverse T2DM [20]. Intuitively, weight loss can ameliorate IR but is daunting to maintain even with intense patient education [27, 28]. Insulin treatment is recommended in patients who have random blood glucose concentrations >250 mg/dL (13.9 mmol/L) and/or HbA1C >9% (75 mmol/mol) [29, 30]. There have been anecdotal observations of pediatric patients with T2DM who recover and achieve glycemic control after starting early insulin therapy [31], although predictors of such clinical success have not been identified.

Therefore, the primary purpose of the proposed study is to describe the characteristics of children who were diagnosed with T2DM and then achieved optimal glycemic control (HbA1C<6.5% on therapy) at the end of one and three years. We also aimed to illustrate the lipid measure outcomes of these children at follow up. We hypothesized that the patients who receive insulin treatment were more likely to achieve optimal glycemic control over time.

## Methods

This was a retrospective chart review of pediatric patients diagnosed with T2DM between 2004–2016 by the Pediatric Endocrinology Division at the Children’s Hospital of Alabama, University of Alabama at Birmingham (UAB). The research protocol was approved by the UAB Institutional Review Board for Human Use. The International Classification of Diseases (ICD-9-CM) diagnosis codes of 250.00 and 250.02 were used to identify all potentially eligible patients with T2DM. The electronic medical records (EMR) of each potentially eligible patient were reviewed to verify the diagnosis of T2DM. Among those with these physician-assigned diagnosis codes, initial inclusion criteria included: HbA1C ≥7.0% at diagnosis, the absence of serum autoimmune markers against islet cell or GAD-65 antigens, BMI >95^th^ centile for age and sex, and elevated C-peptide (above the normal fasting level for the laboratory, ≥2.2 ng/ml) at diagnosis or follow-up. Among this group, only patients who had a follow-up visit between 10 months to 1.5 years after diagnosis were included. American Diabetes Association (ADA) diagnostic criteria for diabetes include a HbA1C of ≥6.5% [32] and, in asymptomatic cases, a repeat HbA1C or fasting glucose is recommended. Due to the retrospective nature of the study, we could not repeat the test and, therefore, only enrolled patients with an HbA1C ≥ 7.0 to increase the specificity of diagnosis [33]. We also collected follow-up information of patients who had a visit between 2.5 and 3.5 years after initial diagnosis when available.

Exclusion criteria included diagnoses of type 1 diabetes (T1DM), maturity onset diabetes of youth, drug induced diabetes, cystic fibrosis-related diabetes, chronic renal or pancreatic disease, Prader-Willi syndrome, documented family history of lipid disorders, or conditions requiring systemic steroid use or immune suppression. We additionally excluded patients with pre-diabetes coded as T2DM (n = 398), patients with HbA1C between 6.5 and 7% (n = 112), and those without follow-up at year 1 (n = 48). Due to the demographics of patients attending the Children’s Hospital, we lacked sufficient sample sizes of Hispanic patients (n = 9) for meaningful comparison and therefore limited our analysis to African American and White patients. Race and ethnicity were ascertained based on parental reports documented in the EMR. For the study purpose, the ‘remission group’ was defined as HbA1C ≤6.5% and being off insulin therapy at follow-up visits. “Optimal glycemic control was defined as achieving HbA1C ≤6.5% at the end of first year based on the treatment target goal as defined by the International Society for Pediatric and Adolescent Diabetes (ISPAD) [30]. ‘Durable glycemic control’ was defined as HbA1C ≤8% as classified in the TODAY trial [20] at follow-up.

All children with T2DM received similar diabetes and nutritional education according to the UAB Endocrine Division protocol and patients were given identical instructions to contact the pediatric endocrinologist frequently for medication adjustments to maintain euglycemia. Insulin treatment was initiated according to the discretion of the attending physician, often dependent on HbA1C levels. Briefly, the management of T2DM in our center is summarized as follows: 1) patients with HbA1C ≥9.0% received oral metformin+ basal, long acting insulin (Glargine/Determir 0.3–0.5 u/kg/day) along with meal bolus and correction factor with rapid acting analogue insulin, (i.e., Lispro and Aspart 0.3–0.5 u/kg/day), 2) patients with HbA1C between 7.5–9% received oral metformin + basal, long acting insulin + rapid acting insulin as correction factor for elevated blood sugars (without meal boluses), and 3) patients with HbA1C <7.5% received metformin alone. None of the patients were using off label medications in the first 3-year period of their diagnosis.

### Statistical analysis

Descriptive statistics were computed for each study variable of interest, including measures of central tendency and dispersion. Because of varying level of compliance with distributional assumptions, we used nonparametric statistical methods for between-group comparisons. Continuous variables are summarized as median [interquartile range]; categorical variables are shown as n (% of those who had data at both visits). All between-group comparisons were conducted with either Wilcoxon signed rank test (for continuous variables) or the Chi-square test (or Fisher’s exact test when needed due to sparse data). To best utilize available repeated measurements, generalized linear mixed models with a binomial link function (i.e. logistic models) were fit to test the effects of insulin therapy on the remission outcome, adjusting for relevant demographic and clinical covariates. Statistical tests were two-sided and were performed using a 5% significance level (i.e. alpha = 0.05). Statistical analyses were performed using SAS software (version 9.4; SAS Institute, Inc., Cary, NC) and R version 3.3.1 (packages ggplot2, dplyr, and lme4).

## Results

**Fig 1:**
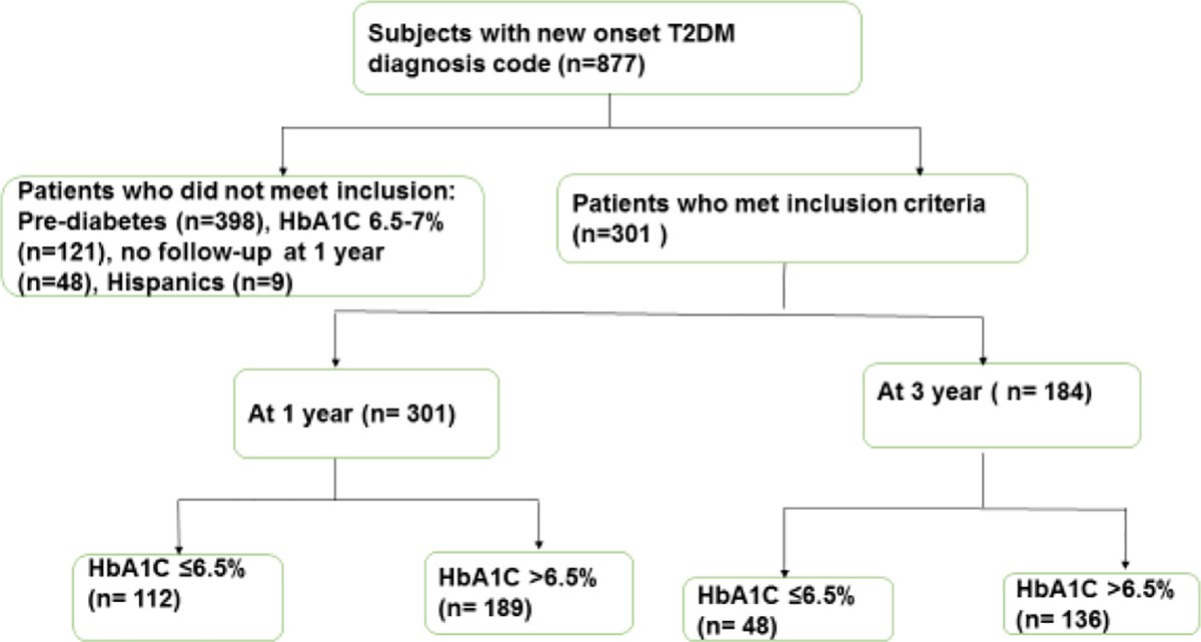
STROBE flowchart. A total of 301 patients met the inclusion criteria.

There were a total of 301 patients who met the inclusion criteria (Fig 1). Table 1 depicts the clinical and laboratory characteristics of the study sample. Among the 301 study participants, the majority were African American (76%) and female (70%), with the median BMI percentile of 99%. Participants were on average obese, and the median HbA1C at diagnosis was 10.6%. There were no significant differences by race in most variables at diagnosis. However, Whites had significantly lower BMI z-scores and higher C-peptide concentrations, total cholesterol: HDL ratio, non-fasting plasma triglycerides (TG), and serum transaminases at diagnosis. A total of 30 patients presented at diagnosis with diabetic ketoacidosis (DKA), and 7 patients presented with documented hyperosmolar hyperglycemic state (HHS). As for treatment, 62.5% initially received insulin and metformin, 15.9% received insulin only (i.e., 78% on insulin treatment) and 20.9% received metformin alone.

**Table 1:** Demographic and clinical characteristics of children with T2DM at the initial diagnosis.

Variable	Total (n = 301)	African American (n = 237)	White (n = 64)	P value
Age (years)	14.0 [3.0]	14.0 [4.0]	14.0 [3.3]	0.18
Females	70.8% (213)	73.8% (175)	59.3% (38)	**0.04**
Weight (kg)	96.0 [33.7]	94.6 [35.0]	97.15 [29.0]	0.37
BMI (kg/m^2^)	35.5 [10.5]	36.3 [10.1]	32.8 [10.7]	**0.02**
BMI z-score	2.4 [0.5]	2.4 [0.5]	2.3 [0.5]	**0.02**
BMI Percentile (%)	99.0 [1.0]	99.0 [1.0]	99.0 [1.0]	0.06
Systolic BP (mmHg)	125.0 [20.5]	124.5 [21.0]	125.0 [22.0]	0.55
Diastolic BP (mmHg)	69.0 [16.0]	69.0 [15.8]	70.0 [15.0]	0.50
HbA1C (%)	10.6 [4.6]	11.0 [4.7]	10.0 [3.9]	0.16
CO_2_ (mmHg)	24.0 [4.0]	24.0 [4.0]	25 [3.0]	0.42
Serum Insulin (mlU/L)	25.8 [40.4]	25.1 [44.9]	42.7 [27.0]	0.29
C-peptide (mg/mL)	3.5 [3.4]	3.2 [3.1]	5.2 [4.4]	**5.3 x 10**^**−6**^
Urine Microalbumin/Cr (mg/gm)	11.2 [26.0]	10.9 [19.8]	28.4 [91.5]	0.08
Total cholesterol * (mg/dL)	174.0 [52.2]	173.5 [51.8]	176.0 [55.0]	0.23
LDL (mg/dL) *	107.0 [53]	104.5 [51.5]	110.5 [47.0]	0.21
HDL (mg/dL) *	35.5 [13.0]	36.0 [13.3]	34.5 [11.0]	0.18
TG (mg/dL)	157.5 [141.2]	139.5 [127.3]	217.5[165.5]	**3.5 x 10**^**−5**^
Non-HDL* (mg/dL)	137.5 [57.8]	134.0 [57.0]	143.0 [51.8]	0.08
TC/HDL ratio*	4.4 [4.3]	4.0 [3.9]	6.5 [6.6]	**0.0001**
ALT (U/L)	28.0 [22.5]	27.0 [19.0]	41.5 [68.0]	**0.0001**
AST (U/L)	27.0 [19.0]	27.0 [16.0]	34.0 [38.0]	**0.009**
Metformin Alone ^α^	63 (20.9%)	49 (20.7%)	14 (21.9%)	0.31
Insulin Alone	48 (15.9%)	34 (14.3%)	14 (21.9%)	
Metformin+Insulin	188 (62.5%)	153 (64.6%)	35 (54.7%)	

Continuous variables are summarized as median [interquartile range]; categorical variables are shown as n (% total, either overall or race-specific).

^α^ There were 2 patients who were not taking any diabetes medications for 6 months after diagnosis

* Abbreviations: T2DM = type 2 diabetes, BP = blood pressure, DKA = diabetic ketoacidosis, BUN = blood urea nitrogen, HbA1C = hemoglobin A1C, HDL = high density lipoprotein cholesterol, LDL = low density lipoprotein cholesterol, AST = aspartate transaminase, ALT = alanine transaminase

Only one patient with T2DM achieved remission (HbA1C≤6.5% off therapy) at the end of one year. The average HbA1C significantly declined from baseline to 1 year, i.e., 10.6 [4.6] vs. 7.3 [3.4], P value 2.2 x 10^−16^. Table 2 illustrates the characteristics of patients by optimal glycemic control at 1-year follow-up. The majority of patients remained on insulin and metformin treatment. At the end of 1 year, 36% of the cohort achieved optimal glycemic control and only 59% of patients achieved durable glycemic control (HbA1C ≤8%).At 1 year, those who achieved optimal glycemic control had improved lipid profiles (LDL, non-HDL, total cholesterol (TC)/HDL ratio); transaminase levels were no longer different between glycemic control groups.

**Table 2:** Follow up characteristics of patients by optimal glycemic control status (i.e. HbA1C ≤ 6.5%) at 1 year after diagnosis.

Variable	Follow-up at 1 year (n = 301)	1 year characteristics A1C ≤ 6.5% at 1 year (n = 112)	1 year characteristics A1C > 6.5% at 1 year (n = 189)	P Value^1^
Age (years)	15.0 [1.6]	15.0 [3.0]	15.0 [3.0]	0.86
Female (n, %)	237 (79%)	71 (63%)	142 (75%)	0.04*
African American (n, %)	237 (79%)	84 (75%)	153 (81%)	0.28
Weight (kg)	101.2 [32.3]	101.3 [31.3]	101.0 [33.2]	0.52
BMI (kg/m^2^)	36.3 [9.5]	35.6 [8.3]	37.0 [10.1]	0.16
BMI z score	2.4 [0.5]	2.3 [0.5]	2.4 [0.5]	0.57
BMI percentile	99.0 [1.0]	99.0 [1.0]	99.0 [1.0]	0.33
Systolic BP (mmHg)	124.0 [19.0]	121.0 [20.0]	126.0 [18.0]	0.18
Diastolic BP (mmHg)	68.0 [12.0]	66.0 [12.0]	69.0 [11.0]	0.06
HbA1C (%)	7.3 [3.4]	5.9 [0.7]	8.9 [3.7]	**<2.2 x 10**^−16^*
CO2 (mmol/L)	26.3 [3.3]	26.0 [3.0]	26.0 [3.0]	0.25
Total cholesterol (mg/dL)	164.0 [47.5]	161.0 [43.0]	167.0 [50.0]	0.05
LDL (mg/dL)	103.5 [44.3]	94.0 [41.0]	108.41 [41.0]	0.03*
HDL (mg/dL)	41.0 [14.0]	41.0 [11.0]	40.0 [15.0]	0.26
Non-HDL (mg/dL)	120.0 [50.0]	116.0 [47.0]	125.0 [54.5]	0.02*
TC/HDL	3.4 [3.4]	2.5 [2.8]	3.8 [4.2]	0.0009*
ALT (U/L)	27.0 [15.0]	26.0 [15.0]	28.0 [14.0]	0.66
AST (U/L)	24.0 [10.0]	23.5 [8.0]	24.0 [11.5]	0.80
Insulin Alone^α^	74 (24.6%)	13 (12%)	24 (13%)	0.003*
Metformin Alone	37 (12.3%)	41 (37%)	33 (17%)	
Insulin & Metformin	186 (61.8%)	56 (50%)	130 (69%)	

* Bold-face typing indicates statistical significance (p<0.05). Continuous variables are summarized as median [interquartile range]; categorical variables are shown as n (% of those who had data at both visits).

^α^There were 3 patients who had exenatide added to their treatment plan at 1 year follow-up and 1 patient not on any medications at follow-up, and were not included for this part of the analysis. One was exenatide and insulin (HbA1C 5.3%), and the other two were only exenatide (HbA1C 6.7% and 7.1%). None of these patients were on exenatide by 3 year follow-up

^1^P value for comparison between ≤6.5% and >6.5% groups at each follow up visit

Table 3 illustrates the characteristics of patients by treatment at diagnosis and 1 year follow up. The insulin treated groups (insulin alone/ insulin + metformin) had higher HbA1C. All treatment groups had statistically significant reductions in the HbA1C with pronounced reductions noted in the insulin alone or insulin + metformin treated groups. The insulin and metformin treated group also had statistically significant improvements in non-HDL, HDL and TC/HDL ratios. Both insulin and metformin treated groups demonstrated improvement in AST.

**Table 3:** Clinical characteristics of patients by treatment group at diagnosis and 1 year follow up.

Variable (units)	Insulin (n = 48)			P value	Insulin + Metformin (n = 188)		P value	Metformin (n = 63)		P value
	At diagnosis		At 1-year follow-up		At diagnosis	At-1 year follow-up		At diagnosis	At 1-year follow-up	
Age (years)	14.0 [5.0]	15.0 [5.0]		0.04*	14.0 [3.0]	15.0 [3.0]	**4.7 x 10**^−6^*	14.0 [4.0]	15.0 [3.5]	0.01*
African American (n, %)	34 (71%)	34 (71%)		n/a	153 (81%)	153 (81%)	n/a	49 (78)	49 (78)	n/a
BMI (kg/m^2^)	31.6 [10.5]	34.4 [8.5]		0.17	36.0 [9.8]	36.9 [9.6]	0.49	37.6 [11.1]	36.5 [9.8]	0.89
BMI z score	2.2 [0.8]	2.3 [0.6]		0.60	2.4 [0.5]	2.4 [0.5]	0.20	2.4 [0.4]	2.4 [0.4]	0.13
BMI percentile (%)	98.5 [2.3]	99.0 [1.0]		0.26	99.0 [1.0]	99.0 [1.0]	0.41	99.0 [0.0]	99.0 [1.0]	0.41
Systolic BP (mmHg)	120.0 [23.5]	122.0 [18.5]		0.79	125.0 [21.0]	124.5 [18.2]	0.34	126.5 [19.0]	125.0 [17.2]	0.91
Diastolic BP (mmHg)	65.0 [17.5]	66.5 [10.0]		0.85	70.0 [15.0]	68.0 [12.0]	**0.01**	68.0 [11.3]	68.0 [11.3]	0.82
HbA1C (%)	12.4 [3.3]	6.8 [3.4]		**2.8 x 10**^−10^*	11.2 [3.8]	7.6 [3.4]	**<2.2 x 10**^−16^*	7.7 [1.5]	7.1 [2.8]	0.001*
Total cholesterol (mg/dL)	171.5 [40.2]	161.0 [42.0]		0.24	174.0 [52.0]	162.5 [46.2]	0.02*	173.0 [46.0]	172.0 [56.7]	0.54
LDL (mg/dL)	107.5 [33.7]	96.0 [32.0]		0.26	107.0 [53.0]	105.0 [42.0]	0.06	100.0 [54.0]	16.5 [45.0]	0.69
HDL (mg/dL)	39.0 [9.5]	36.0 [14.0]		0.89	34.0 [11.0]	42.0 [12.3]	**2.4 x 10**^−8^*	42.0 [17.0]	38.5 [14.0]	0.28
Non-HDL (mg/dL)	139.0 [39.5]	120.0 [41.0]		0.19	138.0 [56.5]	119.0 [50.5]	0.0004*	131.0 [61.0]	132.0 [44.0]	0.75
TC/HDL	4.3 [4.0]	4.4 [3.0]		0.67	4.6 [5.4]	3.1 [2.8]	**1.1 x 10**^−5^*	3.8 [4.4]	3.4 [3.6]	0.94
ALT (U/L)	26.0 [21.0]	26.0 [15.5]		0.99	27.0 [19.8]	28.0 [14.0]	0.67	31.0 [23.0]	27.0 [14.0]	0.08
AST (U/L)	29.0 [37.5]	23.0 [7.5]		0.02*	25.0 [20.0]	23.0 [9.0]	0.06	31.0 [16.0]	24.0 [12.5]	0.01*

* Bold-face typing indicates statistical significance (p<0.05).

A total of 186 patients came back for follow-up at 3-years. Table 4 illustrates the characteristics of patients by optimal glycemic control at 3-year follow-up. Of the patients who had 3 year follow up, the median HbA1C was 9.0% at year 3. Only one patient with T2DM achieved remission (HbA1C≤6.5% off therapy) at 3- year follow-up. At the 3-year follow up, optimal glycemic control was seen in 26% of the patients who came for follow-up at year 3 and 59% had durable glycemic control.

**Table 4:** Characteristics of patients by optimal glycemic control status (i.e. HbA1C < 6.5%) at 3-year follow-up visit.

Variable	Follow-up at 3 year Mean ± SD (n = 184)	A1C ≤6.5% at year 3 Mean ± SD (n = 48)	A1C >6.5% at year 3 Mean ± SD (n = 136)	P Value^1^
Age (years)	15.7±2.2	16.4±2.3	15.5±2.1	**0.04**
Weight	103.4±23.0	102.8±21.1	103.6±22.2	0.84
BMI (kg/m^2^)	37.2±7.2	36.5±8.1	37.4±6.9	0.49
BMI z score	2.3±0.5	2.2±0.7	2.3±0.4	0.15
BMI percentile (%)	97.6±3.8	95.9±6.7	98.1±1.6	**0.03**
Systolic BP (mmHg)	127.4±15.5	129.0±15.8	127.1±15.5	0.45
Diastolic BP (mmHg)	69.0±9.0	68.1±9.4	69.4±8.9	0.42
HbA1C (%)	9.0±2.9	5.9±0.3	10.1±2.5	**0.0001**
BUN (mg/dL)	10.6±2.9	10.4±3.1	10.7±2.9	0.57
Creatinine (mg/dL)	0.64±0.15	0.7±0.2	0.6±0.1	**0.0001**
Total cholesterol (mg/dL)	179.4±46.1	167.3±44.4	183.5±46.3	0.11
LDL (mg/dL)	114.9±41.1	102.0±39.1	117.2±43.2	0.10
HDL (mg/dL)	44.0±12.3	46.9±13.8	42.9±11.7	0.18
Non-HDL (mg/dL)	135.4±45.8	120.4±40.9	140.6±46.7	**0.04**
TC/HDL	4.3±1.5	3.8±1.1	4.5±1.5	**0.008**
ALT (U/L)	31.8±25.8	30.5±28.7	32.0±24.8	0.77
AST (U/L)	25.4±12.6	27.6±11.7	24.3±12.7	0.17
Metformin Alone^α^	37	9	19	0.72
Insulin Alone	19	10	27	
Metformin & Insulin	124	29	88	

^1^ P value for comparison between ≤6.5% and >6.5% groups at follow up visit.

^α^There were 4 patients not taking any diabetes medications at 3- year follow-up.

**Fig 2:**
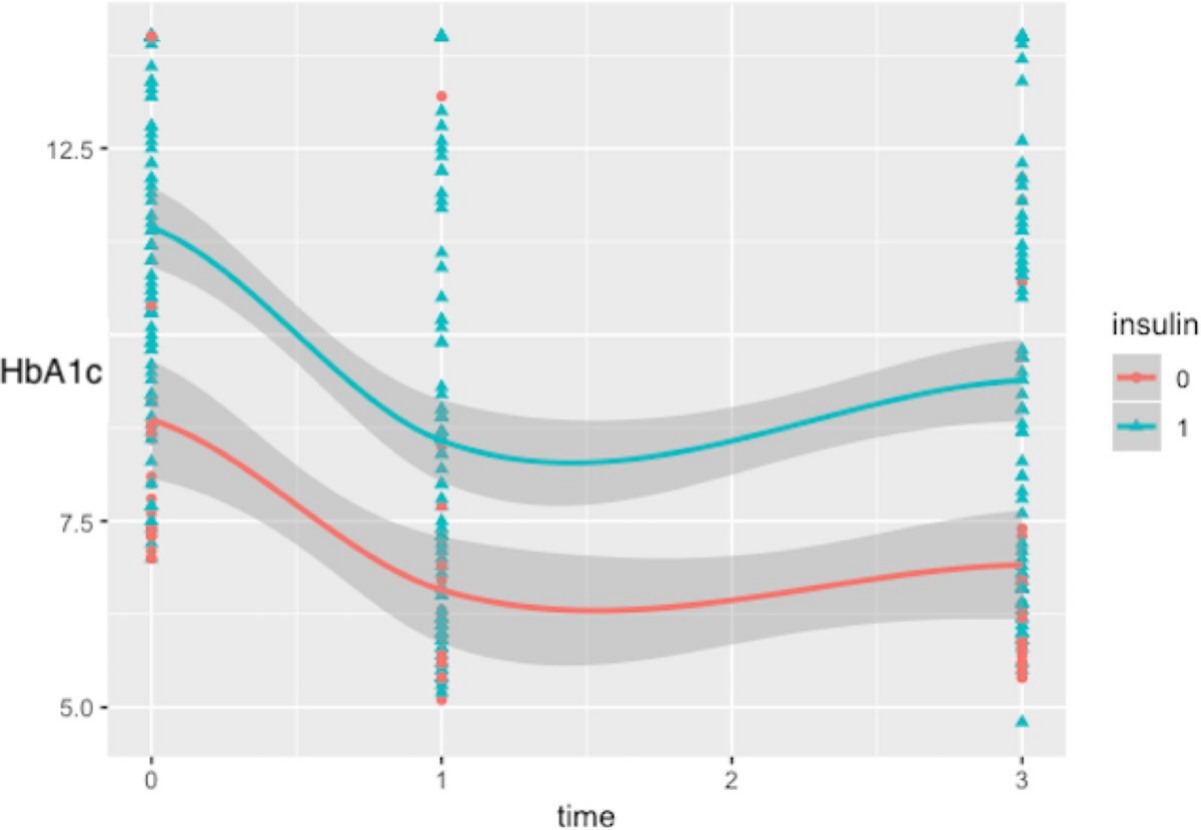
Individual hemoglobin A1C values by treatment over time. Triangles indicate patients treated with insulin at diagnosis (with or without metformin), circles indicate otherwise.

Fig 2 illustrates the individual HbA1C values of patients who continued to follow-up at year 3. Overall trends by group were obtained using the loess smoother in the R ggplot2 package. Patients treated with insulin had significantly higher starting HbA1C, but showed improvements over time similar to the non-insulin treated group.

There were no differences in HbA1C at baseline between patients who came for follow up at year -3 and those who were lost to follow-up. Patients who did not follow up at year 3 were older and heavier, but had otherwise similar clinical covariates.

## Discussion

This study describes the glycemic control and lipid outcomes in children with T2DM who received diabetes care in their home living environment. Only very few patients achieved optimal glycemic control and durable glycemic control at follow-up. Patients who achieved optimal glycemic control and durable glycemic control had significant improvement in lipid parameters, in accordance with findings from other reports [17, 34, 35]. Despite the known salutary effect of insulin treatment in ameliorating the glucotoxicity and lipotoxicity [36], our cohort of children with T2DM were still requiring therapy at the end of year 1[36]. It is likely that only those who required continued therapy came back for follow-up visits, whereas the others who were off therapy were lost for follow-up.

Our patient population is very different from those from the RISE consortium where patients had a baseline HbA1C of 5.7±0.6% [19]. The vast majority (78%) of T2DM patients in our study were treated with insulin alone or insulin coupled with metformin at diagnosis, due to their higher HbA1C at presentation. The patients on insulin treatment alone or in combination with metformin appeared to have marked reductions in HbA1C, likely due to their higher HbA1C at baseline. A plausible explanation for the marked improvement of HbA1C in this group at year 1, is that patients on insulin may perceive their disease as more ‘serious’ compared to those taking a pill and increase compliance with both pharmaceutic and lifestyle interventions. However, over the time of the follow-up, patients treated with insulin showed improvement trajectories similar to those who were not treated with insulin (Fig 2). It is possible that the null findings of the multivariate-adjusted mixed models were due to the small sample size (particularly of the non-insulin treated group). The inability of these patients to come off treatment could be partly explained by the higher HbA1C at diagnosis in the insulin-treated patients, which is likely indicative of significant β-cell failure. Lack of achievement of remission in these patients points towards the deficiencies in the current management approach. The failure of the current therapeutic agents in halting the deterioration of T2DM in children was also observed in youth with recently diagnosed T2DM [19, 37]. The TODAY trial found that metformin monotherapy had higher failure rates compared to the treatment of metformin and rosiglitazone [38], but no comparisons were made with insulin treatment in the TODAY cohort. The TODAY study also demonstrated nearly 50% of glycemic failure in those receiving metformin monotherapy treatment [20, 38]; in comparison, our failure rate was higher (65%).

Further complicating the management is the poor compliance with treatment and lifestyle recommendations so frequently encountered in children with T2DM [3, 12]. Moreover, the improved glycemic control with insulin, notwithstanding the increased weight gain, suggests that it cannot be considered a permanent solution. Of note, the efficacy of prolonged use of insulin has not been tested in T2DM clinical trials in pediatrics [39]. The observations from our study also illustrate the complexity and challenges in the management of children with T2DM. Lack of treatments to address the underlying multiple metabolic defects [13] as well as of an intense, strict lifestyle program, combined with continued weight gain might have accelerated the decline in β-cell function in our population, preventing them from discontinuing insulin therapy.

Similar to other studies, our population was predominantly female and African American [7, 40]. C-peptide levels were higher in White patients at baseline, akin to other studies [41]. Several explanations for these phenomena include: 1) White subjects may have greater β-cell reserve than others, which could enhance their chance of optimal glycemic control from diabetes; 2) White subjects may have been diagnosed earlier than other ethnic groups. Our results demonstrated that obese African American adolescents with T2DM tend to have lower levels of serum TG and lower levels of transaminases compared to Whites, which has been previously reported in other studies [42, 43].

Children with T2DM are known to have atherogenic dyslipidemia [16, 44], due to obesity, chronic insulin resistance and hyperglycemia [45]. Similar to other studies we also found higher LDL (observed at 1-year follow-up) and Non-HDL (observed at 1 and 3- year follow-up) with higher HbA1C [17, 35]. This study indicates that optimal glycemic control would likely result in improved lipid profiles. Among the treatment options, the insulin and metformin treatment resulted in significant improvements in non-HDL, HDL and TC/HDL ratios, highlighting the salutary role of insulin plus metformin treatment for children with T2DM.

Strengths of our study include first and largest study cohort of children with T2DM on insulin treatment and inclusion of a large number of AA subjects, which has to date been a gap in studies of pediatric T2DM. Our findings must be considered in the context of several limitations. This data is from a university-based Children’s Hospital, which caters to the majority of the diabetic patients in the state. However, it is conceivable that patients with relatively milder T2DM from rural areas could have been managed by family doctors and not referred. Because our hospital is a single referral center, the study findings may not be generalizable. Also of concern is selection bias, exacerbated by the inclusion of only patients with at least a one year follow up visit. The three-year follow-up data was only available in 62% of patients which limits the ability to accurately interpret the outcome data at year three. There is a risk for selection bias since it is possible that only those who had severe disease came back for follow-up. Given the high dropout of the medical care system seen in pediatric T2DM[3], many patients were lost to follow-up and may have differed in their disease risk and characteristics from those included in the study. Due to the retrospective nature of the study, we were unable to assess the compliance of patients with different treatment regimens. Moreover, the treatments were not randomly assigned which precludes causal comparison of outcomes. Also, we do not have c-peptide data on all the patients to assess the β-cell reserve. Noncompliance with the treatment regimen is a well-known reason for poor outcome in children with T2DM [3, 12, 46]. We used an inclusion criterion for HbA1C of ≥ 7.0%, which may have excluded some mild, early onset T2DM and might have resulted in depiction of larger number of patients not having remission. Also, we could not adjust for confounders such as physical activity, lifestyle modifications, socioeconomic factors, and most importantly, compliance with prescribed treatment. Furthermore, lack of data on auto antibodies other than islet cell or GAD-65 antigens, might have resulted in inadvertent inclusion of some adolescents with T1DM. Finally, our study is vulnerable to the issues that commonly plague observational studies, including the difficulties of establishing causal relationships beyond mere associations.

Because young patients are at significantly higher risk to develop complications from T2DM[6] it is imperative to establish the most tolerable, least taxing and most efficacious treatment. On balance, current evidence highlights the need for a paradigm shift to preserve β-cell function in the management of T2DM in children.

## Conclusions

Most children with T2DM required continued treatment at follow-up. The currently available treatment options do not sufficiently reduce HbA1C to healthier levels or achieve long-term remission.

## Supporting information

S1 Dataset (XLSX)
